# Measuring brain potentials of imagination linked to physiological needs and motivational states

**DOI:** 10.3389/fnhum.2023.1146789

**Published:** 2023-03-15

**Authors:** A. M. Proverbio, F. Pischedda

**Affiliations:** Laboratory of Cognitive Electrophysiology, Department of Psychology, University of Milano-Bicocca, Milan, Italy

**Keywords:** EEG/ERP, mental imagery, motivational states, ERP markers, brain computer interface (BCI)

## Abstract

**Introduction:**

While EEG signals reflecting motor and perceptual imagery are effectively used in brain computer interface (BCI) contexts, little is known about possible indices of motivational states. In the present study, electrophysiological markers of imagined motivational states, such as craves and desires were investigated.

**Methods:**

Event-related potentials (ERPs) were recorded in 31 participants during perception and imagery elicited by the presentation of 360 pictograms. Twelve micro-categories of needs, subdivided into four macro-categories, were considered as most relevant for a possible BCI usage, namely: primary visceral needs (e.g., hunger, linked to desire of food); somatosensory thermal and pain sensations (e.g., cold, linked to desire of warm), affective states (e.g., fear: linked to desire of reassurance) and secondary needs (e.g., desire to exercise or listen to music). Anterior N400 and centroparietal late positive potential (LPP) were measured and statistically analyzed.

**Results:**

N400 and LPP were differentially sensitive to the various volition stats, depending on their sensory, emotional and motivational poignancy. N400 was larger to imagined positive appetitive states (e.g., play, cheerfulness) than negative ones (sadness or fear). In addition, N400 was of greater amplitude during imagery of thermal and nociceptive sensations than other motivational or visceral states. Source reconstruction of electromagnetic dipoles showed the activation of sensorimotor areas and cerebellum for movement imagery, and of auditory and superior frontal areas for music imagery.

**Discussion:**

Overall, ERPs were smaller and more anteriorly distributed during imagery than perception, but showed some similarity in terms of lateralization, distribution, and category response, thus indicating some overlap in neural processing, as also demonstrated by correlation analyses. In general, anterior frontal N400 provided clear markers of subjects’ physiological needs and motivational states, especially cold, pain, and fear (but also sadness, the urgency to move, etc.), than can signal life-threatening conditions. It is concluded that ERP markers might potentially allow the reconstruction of mental representations related to various motivational states through BCI systems.

## Graphical Abstract

Expert classification of EEG signals linked to imagined physiological needs and motivational states, based on ERP markers. 
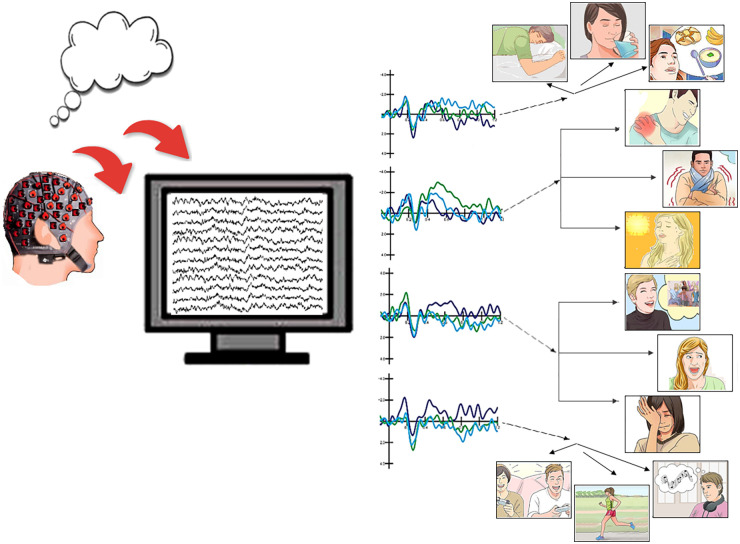

## 1. Introduction

Clear evidences have been provided of reliable electrophysiological markers for motor imagery (e.g., Milanés-Hermosilla et al., 2021; Mattioli et al., 2022), and, to a lesser extent, perceptual and cognitive imagery (Cai et al., 2013; Leoni et al., 2021, 2022; Proverbio et al., 2022), for communication with brain computer interface (BCI) systems (Ash and Benson, 2018). In contrast, not much is known about the electrophysiological markers of motivational imagery (craves, wills, needs and desires), despite the fact that this particular type of mental content is valuable for interacting with patients with disorders of consciousness, such as coma or locked-in syndrome. According to some studies (Kavanagh et al., 2005) imagination would be able to activate measurable responses to visceral desires, qualitatively similar to emotional ones, regardless of whether the stimulus is perceived or imagined. For example, mental imagery of food-related stimuli was found to modify the salivation reflex and even increase or decrease salivary pH levels during tasks whose instructions were to imagine eating a lemon and drinking milk, respectively (Vanhaudenhuyse et al., 2007). These studies, conducted in the field of BCI, may prove useful in demonstrating the presence of consciousness in patients with Locked-in Syndrome, who could not otherwise communicate their still-present consciousness. In this study it was explored the possibility to record and identify reliable electrical markers of inner motivational and physiological states associated to specific desires/needs. We investigated a variety of “craves,” including desiring a change in the environmental conditions (hot, cold), in the person’s homeostatic balance (thirst, hunger, sleep), desiring pain relief, psychological comfort, social contacts, movement, music entertainment, social recreation. While fMRI measures of food or drug craving were previously reported, as well as of thermal, pain or visceral sensations, not much knowledge is available about possible ERP indicators of motivational states (“mind reading” approach). According to Kavanagh et al. (2005) imagery would be a key characteristic of desire: developing mental images of the target object or action of desires/needs would play a key role in the experience. It can be hypothesized that the evocation of motivation-related imagery activates areas similar to those activated in similar types of imagery, since they relate to the same domain as the imagined motivational state or need (Belardinelli et al., 2004). One might expect, for example, activation referable to gustatory imagery during imagining the desire for food (i.e., primary gustatory cortex, including bilateral frontal operculum and bilateral anterior insula, McNorgan, 2012); or activation of areas involved in auditory imagery during the simulation of the desire to listen to music (planum temporale, McNorgan, 2012). We hypothesized that since “craving” corresponds to imagining the desired state, as demonstrated in addicted individuals craving for drug (Bradley and Moorey, 1988), it was perhaps possible to study motivational states through their imagination/simulation.

ERP studies on electrophysiological indices of motivational states are rather scarce. Suess and Abdel Rahman (2015) studied ERP components in a mental imagination task of emotionally characterized faces while also comparing them with a perception condition. In both cases, a late positive potential (LPP) modulated by the emotional content of the stimuli was shown to be more anterior in imagination and more posterior in perception. Consistently, Marmolejo-Ramos et al. (2015) found similarity between ERP components related to perception and imagination of affectively charged stimuli. In this study, participants were exposed to images of food or to pleasant, unpleasant and neutral images; they were then asked to create a visual mental image of the stimuli (imagery condition). The results showed that in the imagery condition there were activations corresponding to the motivational sensorimotor and parietal systems, defensive vs. appetitive, according to the stimulus appeal. Those systems were activated to different extents, producing a graded pattern of posterior negativity and late positive responses that were more prominent for unpleasant than pleasant food. LPP was larger during perception than imagination and it was significantly reduced during imagery of unpleasant photos, suggesting inhibition of undesired memories. In fact, according to the authors, the LPP response, a robust component of emotional processing (MacNamara, 2018), might be related to the reenactment of negative images and the suppression of unpleasant and painful meanings (Brown et al., 2012).

The aim of the present study was to investigate, through EEG/ERP recording, the existence of distinctive ERP components related to mental imagery processes. Specifically, the imagination of physiological and motivational states such as visceral and primary needs (hunger, thirst, sleep), somatosensory sensations (hot, cold, pain), affective states (joy, fear, and sadness), and secondary needs (the desire to listen to music, exercise, and play with friends) were investigated. The various needs might be grouped into two supra-ordinal classes, depending on their more or less volitional or irrepressible nature, as summarized in the paper’s title. “Motivational states” included the desire to play with friends, listening to music, exercising, sharing emotions and being comforted from fear and sadness. Imagined “physiological needs” included more visceral and uncontrollable urges, relative to homeostatic regulation of bodily physiological functions (control of temperature, pain level, food or drink income, and sleep).

To elicit imaginative/motivational states, participants were visually shown tables from a previously validated Pictionary and their perceptual signals were recorded and compared with imaginative ones. Based on previous literature, ERP components were expected to be larger in perceptual modalities than in imaginative modalities (Marmolejo-Ramos et al., 2015; Suess and Abdel Rahman, 2015). Late anterior positivity (LPP) was expected to be sensitive to class categories, while being smaller to negative and painful subjective experiences (Brown et al., 2012; Marmolejo-Ramos et al., 2015).

## 2. Materials and methods

### 2.1. Participants

Thirty-one participants (14 males, 17 females), aged 18 to 35 years (23.26, SE = 2.73; schooling = 16.65 years, SE = 1.68), took part in the EEG study. They had normal or corrected vision, had no current or previous neurological or psychiatric disorders, and were not taking psychotropic drugs or substances that could affect brain activity. All participants were right-handed, as assessed through the Edinburgh inventory questionnaire. Students were recruited through the Sona-system and received credit for their participation. Eleven participants were excluded from the final sample due to excessive EEG/EOG artifacts. The final sample thus included 20 participants (8 males, 12 females), aged 18–35 years (23.20, SE = 1.70), right-handed (dominance score = 0.84, SD = 0.17) and schooling = 16.80, SE = 1.58. A questionnaire designed to measure the ease/difficulty with which subjects had been able to imagine the different categories of needs was also administered after EEG recording. Participants were asked to rate, on a 5-point Likert scale (from 1 = very difficult to 5 = very easy), the imageability of the different macro-categories of stimuli. Each participant provided written informed consent. The experiment was conducted in accordance with international ethical standards and was approved by the Ethics Committee (protocol no: RM-2020-242).

### 2.2. Stimuli

**FIGURE 1:**
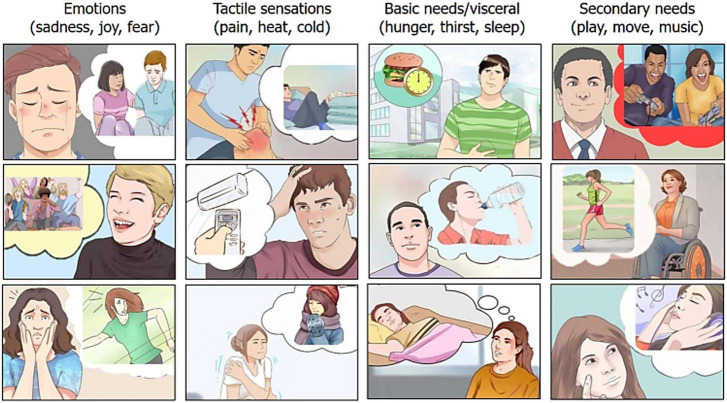
Example of pictograms related to the 12 motivational states or craves. Taken from the BCI pictionary by Proverbio and Pischedda (2023).

Stimuli were taken from a validated Motivational Pictionary (Proverbio and Pischedda, 2023) with 60 color plates and depicting male and female adults expressing 12 different motivational states (see Figure 1 for some examples).

Each micro-category was illustrated by five different pictograms (variants), in which the same motivational state was illustrated but in a different way, for improving generalizability of the main need. For example, for “feeling hot” pictograms depicted: the desire to be sprayed with ice water, to eat a popsicle, to turn on the air conditioning, to put an icy cloth on the forehead, to stand in front of a fan. Validation demonstrated the strong communicative validity of the illustrations (rated on average 2.7, on a 0–3 scale), with an accuracy of 98.4%. Motivations and craves expressed by stimuli could be subdivided into four macro categories that were: *Primary needs* for maintaining homeostasis, mediated by visceral sensations, such as hunger, thirst and sleep. These visceral sensations would enable physiological regulation to maintain adaptive set points required for the integrity of the organism (Tucker and Luu, 2021) and would be based on the limbic system Papetz circuits. According to Mac Lean they would represent primary survival needs. *Somatosensory sensations*, such as cold, hot and pain, mediated by peripheral nociceptors and processed by the somatosensory system, signaling harmful thermal or physiological conditions (Gracheva and Bagriantsev, 2021) and the need to reverse the unpleasant condition. *Affective states*, such as fear, sadness, cheerfulness, represented both at cortical and subcortical level (Phillips et al., 2003). Each emotion would have its own dedicated neural circuitry, architecturally distinct. Very generally, cheerfulness would be associated (among other areas) to the ventromedial mesolimbic dopaminergic system, sadness to the abenula and the cingulate cortex, and fear to the amygdala nuclei (e.g., Gu et al., 2019). *Secondary needs* (desires or motivational states), such as the desire for music listening, moving (running, jumping, dancing), playing video-games with friends. They can be assimilated to wishes, and were selected taking into account the most common and sought-after activities among young people. They are reward-related hedonic experience, but, unlike the other needs, sensation and states (Paulus, 2007), they can be inhibited and voluntary suppressed/postponed, which imply a neocortical representation.

Each plate represented a character, of the apparent age of a student, in a specific emotional and motivational state; a little cloud illustrated the circumstantial need, desire or state of the character. For example, if the visible state was freezing, the represented desire was for warmth, as described in Table 1. While for primary and secondary needs, and for somatosensory sensations, the needs were clearly defined based on the motivational state (e.g., need of food for hunger, need of sleep for sleep, need of exercise for movement, etc.) for affective states, the needs (shown in Table 2) were established based on prior assessment (see Proverbio and Pischedda, 2023 for details). Stimuli were equiluminant as assessed through a Minolta luminance meter. Luminance values were subjected to repeated measures ANOVAs. Analyses yielded no significant differences between micro-categories [*F*(11,44) = 0.41, *p* = 0.94] and macro-categories [*F*(3,12) = 0.21, *p* = 0.89]. Mean luminance values within macro-categories were: 71.40 cd/m2 for Primary needs, 74.50 cd/m2 for Secondary needs, 70.25 cd/m2 for Somatosensory Sensations, and 70.27 cd/m2 for Affective states.

**TABLE 1:** The 12 motivational states, grouped according to their content and nature, and ordered as a function of their imageability.

Motivational states	Imageability
**Primary needs (visceral)**	**(*M* = 4.45, SE = 0.11)**
I am hungry – I need food	
I am thirsty – I need to drink	
I am sleepy – I need to sleep	
**Somatosensory sensations**	**(*M* = 3.65, SE = 0.18)**
I feel cold – I need warmth	
I feel hot – I need cool	
I feel pain – I need pain relief	
**Affective states**	**(*M* = 3.25, SE = 0.22)**
I am sad – I need consolation	
I am cheerful – I need to communicate	
I am scared – I need reassurance	
**Secondary needs (desires)**	**(*M* = 3.20, SE = 0.23)**
I feel like listening to music – I need music	
I feel like moving – I need movement	
I feel like playing – I need to play	

**TABLE 2:** Summary of the main content reported through the questionnaire, with the aim of determining which motivational aspects were related to certain emotional states.

Motivational state	Desire (cloud’s content)
I feel fear	Physical and emotional proximity to someone; being in a safe/familiar place; escape
I feel pain	Let the pain go away; to be medicated; to feel safe or have support
I feel sadness	Carry out favorite activities; support/companionship of loved ones; crying/burdening
I am cheerful	Sharing happiness with loved ones; expressing affection/hugging; expressing joy (jumping, dancing, celebrating)

These responses were used to create the content of the clouds for these micro-categories of stimuli.

### 2.3. Procedure

**FIGURE 2:**
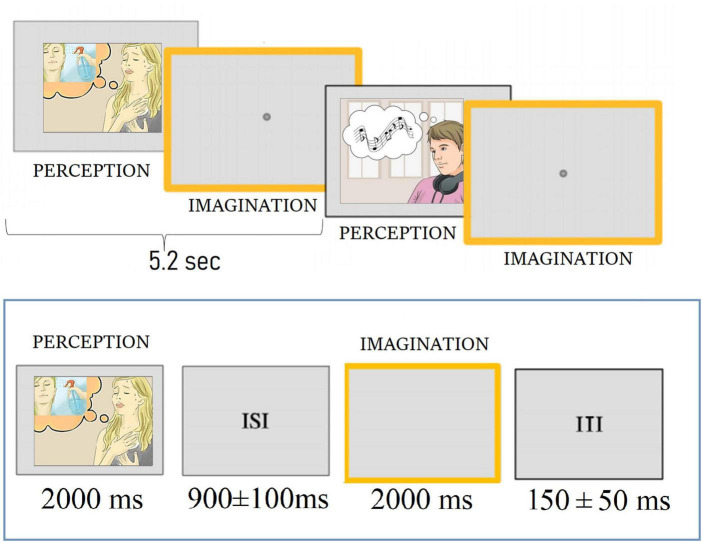
Time sketch of the experimental procedure.

In total, 360 stimuli (60 original stimuli, repeated 6 times each) were presented in a randomized manner. The presentation of stimuli was subdivided into 10 runs comprising 36 stimuli each. Stimuli lasted 2000 ms and were followed by an ISI (empty bright screen) ranging from 900 ± 100 ms, also meant to cancel possible retinal after-images related to the previous stimulation. A visual *probe* prompting imagery (a bright yellow frame on a gray background cornering the screen) lasting 2000 ms followed. The Inter Trial Interval (ITI) was 150 ± 50 ms (see Figure 2). Stimuli were 640 × 480 pixels in size (13.5 × 9.5 cm) and subtended 6° 47′ 16′′ × 4° 46′ 37′′ of visual angle. The PC screen was located at a distance of 114 cm from the observer’s eyes, and a fixation point had to be maintained throughout the recording. Written instructions were provided to participants: *“When the yellow frame appears, imagine the motivational, emotional state or feeling, to which the image you have just seen refers, reconstructing it in your mind as vividly as possible, always keeping your gaze fixed on the center of the screen. The feeling you are trying to evoke must be subjective, personal, centered on your body and the way you experience the sensations. The image is just one example. For example, if you have to imagine being hungry, imagine a strong desire for the foods you crave now (e.g., some pasta) not necessarily those illustrated in the image. Try to reconstruct your visceral feelings.”* To familiarize participants with setting and task a brief training was given before EEG recording, based on the presentation of two runs of 15 stimuli each, selected from those discarded during validation phase. The task consisted in visualizing (in the perception condition), and internally generating the motivational state indicated (in the imagination condition), as accurately as vividly as possible.

In order to maintain high levels of attention toward stimulation, subjects were warned that at the end of the session they would have to answer questions about the content and nature of imagined states. The questionnaire, actually comprised 4 questions (1 for each stimulus category), in which participants were asked to assess the imagination easiness or difficulty by means of a 5-point Likert scale, where “1” corresponded to very difficult and “5” corresponded to very easy. The question (declined for the various stimuli) was: ≪Thinking back to the exercise of mentally simulating the various motivational and physiological states, what was it like to imagine …?>>.

### 2.4. EEG recording and analysis

Brain activity was recorded from 128 electrode sites located according to the 10-5 International system (Oostenveld and Praamstra, 2001). Horizontal (hEOG) and vertical (vEOG) electro-oculograms were also recorded. Averaged mastoids served as the reference lead. Electrode impedance was kept below 5 kΩ. Sampling rate was 512 Hz. The EEG and EOG signals were recorded using *Cognitrace system* (ANT Software) and were amplified with a bandpass filter (0.016–70 Hz). EEG artifacts exceeding ± 50 μV were automatically rejected before averaging. EEG epochs, synchronized with stimulus presentation, were processed through *EEProbe system* (ANT Software). ERPs were averaged offline from 100 before to 1200 ms after stimulus onset. Separate sets of ERP waveforms were computed in response to pictograms and imagery probes, in the perception and in the imagination conditions. ERPs were temporally synchronized with the onset of stimuli, i.e., the pictograms (in the perception condition) and the empty yellow frames (in the imagination condition). ERP components were identified and measured with respect to the average baseline voltage recorded in the −100/0 ms pre-stimulus interval. Four swLORETAs (*standardized weighted low-resolution electromagnetic tomographies*; Palmero-Soler et al., 2007) were computed on N400 and LPP potentials measured during imagination of secondary needs (Music and Movement), that most correlated with imageability scores.

Three types of repeated-measure ANOVAs were performed: (i) to compare all macro-categories during perception vs. imagination conditions. (ii) To compare different micro-categories during perception vs. imagination conditions. (iii) To compare ERP amplitude values across all microstates during the imagery condition, for BCI purposes. An anterior negativity (N400) was quantified within the 400–600 ms latency range in both the perception and imaginative conditions at anterior/frontal, prefrontal and fronto/central sites. A late posterior positivity (LPP) was quantified within 600–800 ms in perception condition, and 800–1000 ms in the imagination condition, at central, parietal and occipital sites. Components were quantified when in time, and where at scalp they reached their maximum amplitude, and based on previous literature on imagery-related components [e.g., the fronto-polar N400 (Schendan and Ganis, 2012) and centro/parietal LPC (Suess and Abdel Rahman, 2015)]. For both components, ANOVA factors were: Macro-category (needs, somatosensory sensations, affective states, and secondary needs), Electrode (3 levels) and Hemisphere (left, right). For micro-states factors were: Condition (Perception, Imagination), Category (3 levels, depending on the macro-category), electrode (4 levels) and hemisphere. The ANOVAs performed on the 12 microstates for the N400 and LPP data had 2 factors of variance: Micro-category (12 levels) and Hemisphere (left, right), and were performed on N400 values recorded at AF3 and AF4 sites, and LPP values recorded at C1, C2 sites. A correlation analysis (Spearman’s Rho) was then carried out between the mean amplitude values of each subject’s N400 and LPP components with the individual imageability values related to the 4 macro-categories, which emerged from the ease of imagining questionnaire. Finally, a standardized weighted LORETA was conducted to investigate neural activations related to motivational states belonging to the secondary needs, for which it was found a correlation with imageability. A more in-depth and comprehensive analysis of the 12 motivational states will be conducted in an independent study.

## 3. Results

### 3.1. Imageability scores

The imaginability scores attributed to the images belonging to the various macrocategories (on a 5-point Likert scale, from 1 = very difficult to 5 = very easy) were subjected to a 4-way ANOVA whose variance factor was Macrocategory, with 4 levels (primary and secondary needs, somatosensory sensations, affective states). The results showed that, on average, all motivational states were fairly easy to imagine, with mean scores ranging from a maximum of 4.45 (“very easy”) for primary needs to a minimum of 3.20 (“easy”) for secondary needs. The main factor macro-category factor was statistically significant [*F*(3, 57) = 9.12, *p* < 0.001]. Tukey’s *post hoc* comparisons revealed a significant difference between imageability of primary needs vs. all other macro-categories (*p* < 0.001 vs. secondary needs and affective state, and *p* < 0.05 vs. somatosensory sensations (see Table 1 for mean values and standard errors).

### 3.2. ERP results: Macro-category analyses

**FIGURE 3:**
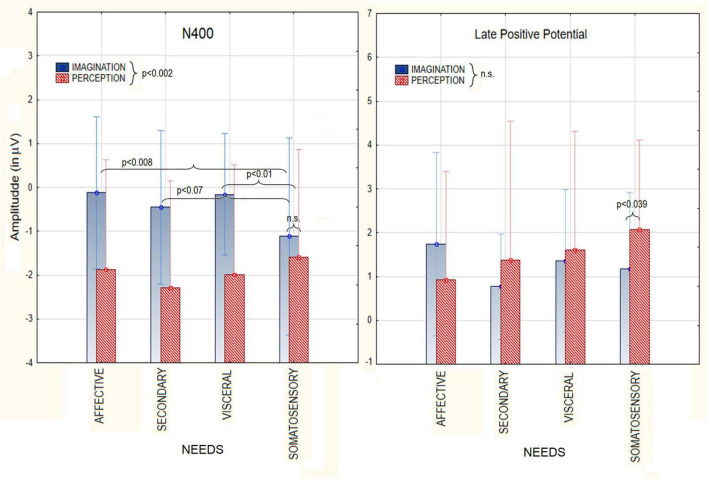
Mean area amplitude values of N400 component **(Left)** and LPP **(Right)** as recorded during the perception and imagination conditions, as a function of the macro-category of needs.

The ANOVA relative to N400 amplitudes showed a significant effect of Condition [*F*(1, 19) = 12.98 *p* < 0.002), with larger amplitudes during perception (−1.93 μV, SE = 0.28) than imagination (−0.46 μV, SE = 0.41), as visible in Figure 3. The ANOVA also yielded the significant interaction of Condition × Category [*F*(3, 57) = 26.58, *p* < 0.03]. Tukey *post-hoc* tests revealed that N400 was always larger during perception than imagination, except that for somatosensory sensations (pain, hot and cold), to which responses were of comparable amplitude across conditions. Only in the imagination condition, N400 amplitudes measured in response to stimuli belonging to the somatosensory macro-category (−1.11 μV, SE = 0.43) were significantly greater than in response to affective states (−0.12 μV, SE = 0.33) and visceral needs (−0.15 μV, SE = 0.26). The significant effect of Electrode [*F*(2, 38) = 18.52, *p* < 0.0001] showed larger N400 responses at anterior frontal sites (AFp3h, AFp4) regardless of condition or stimulus category.

The ANOVA relative to LPP amplitudes showed no main effect of Condition (*p* = 0.57). However the interaction of Condition × Category [*F*(3,57) = 3.42, *p* < 0.02], and relative *post-hoc* tests, showed that LPP was larger in the perceptual than imaginative condition in response to somatosensory sensations. The further interaction of Condition × Electrode [*F*(2,38) = 12.72, *p* < 0.0001] indicated much larger LPPs in the perceptual than imaginative conditions at parietal sites (P7, P8). LPPs were larger at central sites (C1,C2) during imagination and at parietal sites during perception. Finally, the significant interaction of Category × Hemisphere [*F*(3,57) = 5.95, *p* < 0.001] showed that, regardless of condition, LPP was larger in amplitude over the right than left hemisphere for all macro-categories except somatosensory sensations (hot, cold, pain).

### 3.3. ERP results: Micro-category analyses (perception vs. imagination)

#### 3.3.1. Mean area amplitude of N400

##### 3.3.1.1. Primary visceral needs (feeling hungry thirsty and sleepy)

**FIGURE 4:**
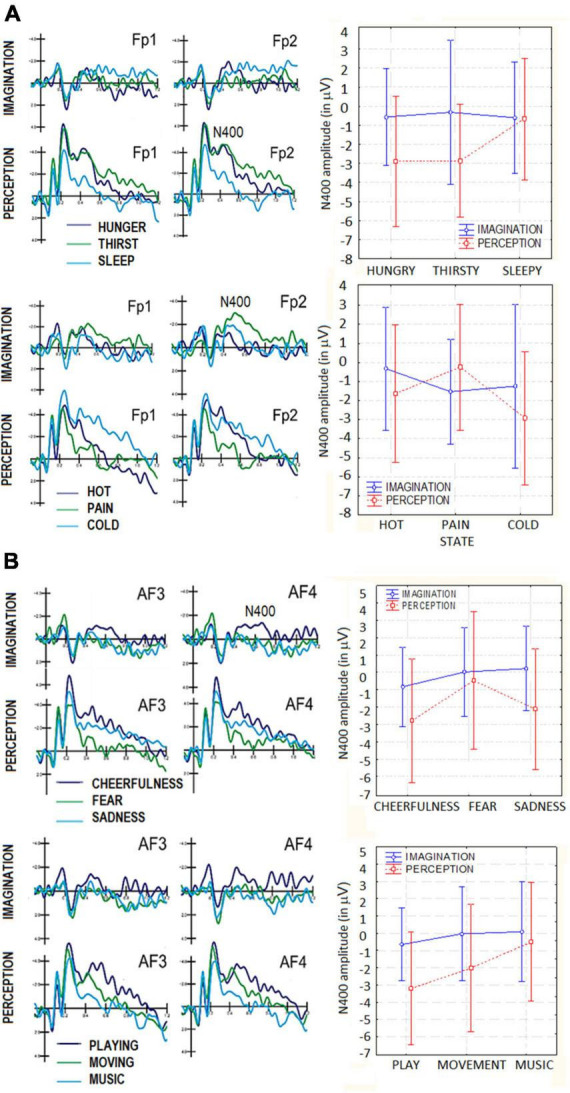
**(A)** Grand-average ERP waveforms recorded at anterior scalp sites in the perceptual and imaginative conditions, as a function of motivational states. Top = primary needs; bottom = somatosensory sensations: **(B)** grand-average ERP waveforms recorded at anterior scalo sites in the perceptual and imaginative consition, as a function of motivational states. Top = affective states; bottom = secondary needs.

The ANOVA performed on N400 amplitudes showed the significance of Condition factor [*F*(1,19) = 13.07, *p* < 0.002] with larger amplitudes in the perceptual (−2.13 μV, SE = 0.44) than imaginative condition (−0.49 μV, SE = 0.34). The significance of Category factor [*F*(2,38) = 3.78, *p* < 0.03] showed that N400 was larger to hunger (−1.72 μV, SE = 0.41) than sleep category (−0.63 μV, SE = 0.39). The interaction between Category × Condition was statistically significant [*F*(2,38) = 4.94, *p* < 0.01]. *Post-hoc* tests showed that, while there was no difference between perception and imagery for Sleep category, N400 was greater in the perception condition for hunger (−2.89 μV, SE = 0.57) and thirst (−2.86 μV, SE = 0.49) categories, which differed from the sleep condition (−0.66 μV, SE = 0.53). In the imaginative condition this difference was not significant (*p* = 1.00 and *p* = 0.99). The significance of electrode factor [*F*(3,57) = 9.13, *p* < 0.0001] showed larger N400 responses at prefrontal (FP1-FP2 = −1.75 μV, SE = 0.32) than other sites. ERP waveforms relative to N400 data are shown in Figure 4A.

##### 3.3.1.2. Somatosensory sensations (feeling cold, hot, pain)

The ANOVA performed on N400 amplitudes did not yield the significance of factor condition *per se*, with no difference between the perceptual and imaginary condition (*p* = 0.32) The Category factor was significant [*F*(2,38) = 4.23, *p* < 0.02]. *Post hoc* comparisons showed larger N400 amplitudes to cold (−2.08 μV, SE = 0.52) than hot category (−0.88 μV, SE = 0.33). The significant interaction of Condition × Category [*F*(2,38) = 7.70, *p* < 0.001] showed that in the imagery condition N400s to cold and pain sensation did not differ (*p* = 0.95), while they did in the perceptual condition (cold = −2.93 μV, SE = 0.59; pain = −0.24 μV, SE = 0.59). The significance of the electrode factor [*F*(3,57) = 8.52, *p* < 0.0001] showed larger N400 responses at FP1-FP2 (*M* = −1.80 μV, SE = 0.46) than other sites.

##### 3.3.1.3. Affective states (feeling sadness, cheerfulness, fear)

The ANOVA performed on N400 amplitudes showed the significance of Condition factor [*F*(1,19) = 9.45, *p* < 0.005], with larger amplitudes in the perceptual (−1.79 μV, SE = 0.47) than imaginative condition (−0.19 μV, SE = 0.31), as shown in Figure 4B. The factor Category was also significant [*F*(2,38) = 6.65, *p* < 0.003]. Tukey *post hoc* comparisons showed that N400 to cheerfulness (−1.80 μV, SE = 0.32) was larger than that to fear stimulus category (−0.22 μV, SE = 0.44), regardless of sensory condition (imagination vs. perception). The significance of electrode factor [*F*(3,57) = 13.43, *p* < 0.001] showed larger N400 responses at AF3-AF4 (−1.14 μV, SE = 0.30) and FP1-FP2 (−1.50 μV, SE = 0.32) than other sites.

##### 3.3.1.4. Secondary needs (music listening, moving, and playing)

The ANOVA performed on N400 amplitudes yielded the significance of Condition [*F*(1,19) = 11.13, *p* < 0.003], with larger amplitudes in the perceptual (−1.90 μV, SE = 0.50) than imaginative condition (−0.20 μV, SE = 0.27), as shown in Figure 4B. The Category factor was also significant [*F*(2,38) = 9.15, *p* < 0.0006). Tukey *post hoc* comparisons showed larger N400 responses to playing (−1.92 μV, SE = 0.35) than music listening (−0.21 μV, SE = 0.37) categories, for both imagination and perception condition. Additionally, the significant interaction of Condition × Category [*F*(2,38) = 3.31, *p* < 0.045], and relative *post hoc* comparisons, showed that, in both sensory conditions, N400 was larger to playing (perception = −3.19 μV, SE = 0.55; imagery = −0.65 μV, SE = 0.35) than moving (perception = −2. 00 μV, SE = 0.62; imagery = −0.03 μV, SE = 0.45). Only in the perceptual condition N400 was larger to moving than music listening categories (perception = −0.51 μV, SE = 0.58, imagery = 0.08 μV, SE = 0.48). In the perception condition, N400 measured in response to playing category was significantly greater than that measured in response to music listening category (*p* < 0.001), while in the imagery condition this difference was not significant (*p* = 0.76). The significance of electrode (*F*(3,57) = 8.67, *p* < 0.0001) showed larger N400 responses over prefrontal (FP1-FP2 = −1.48 μV, SE = 0.37) and anterior frontal AF3-AF4 (−1.19 μV, DS = 0.28) than other sites.

#### 3.3.2. Mean area amplitude of late positive potential (LPP)

##### 3.3.2.1. Primary visceral needs (feeling hungry thirsty and sleep)

**FIGURE 5:**
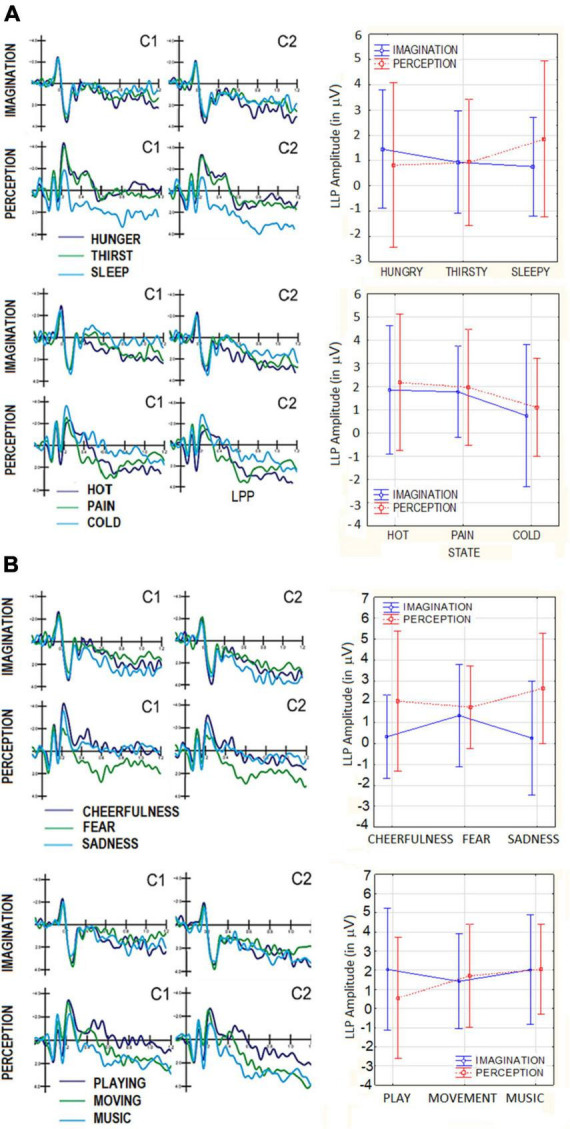
**(A)** Grand-average ERP waveforms recorded at central and posterior scalp sites in the perceptual and imaginative conditions, as a function of motivational states. top = primary needs; bottom = somatosensory sensations: **(B)** grand-average ERP waveforms recorded at central and posterior scalp sites in the perceptual and imaginative conditions, as a function of motivational states. Top = affective states; bottom = secondary needs.

The ANOVA performed on LPP amplitudes yielded the significant interaction of category × electrode [*F*(2,38) = 4.65, *p* < 0.01]. *Post hoc* showed that at central sites (C1, C2) LP was larger to sleep (1.92 μV, SE = 0.54) than thirst images (1.03 μV, SE = 0.54). Additionally LP was larger to hunger (0.86 μV, SE = 0.41) than sleep images (0.67 μV, SE = 0.42) at occipital sites (O1,O2). ERP waveforms relative to LPP component are visible in Figure 5A. No significant effect of Condition was found (*p* = 0.83). However the interaction of Condition × Category × Electrode factors reached significance [*F*(2,38) = 7.11, *p* < 0.002], with larger LPP responses in the perceptual than imagery condition, at central sites, where LPP was of greater amplitude to sleep (2.71 μV, SE = 0.89) than hunger (0.95 μV, SE = 0.79) and thirst (0.70 μV, SE = 0.66) images. LP was larger at scalp over the right (1.46 μV, SE = 0.44) than left hemisphere (0.77 μV, SE = 0.44), regardless of Category, as shown by the hemisphere factor’s significance [*F*(1,19) = 5.81, *p* < 0.03].

##### 3.3.2.2. Somatosensory sensations (feeling cold, hot, pain)

This ANOVA yielded the significance of Category × Electrode [*F*(2,38) = 7.12, *p* < 0.002], with larger amplitudes to pain (2.43 μV, SE = 0.43) than cold images (0.96 μV, SE = 0.54) at central sites (C1,C2 = *p* < 0.001). The interaction of Condition × Category × Electrode [*F*(2,38) = 3.43, *p* < 0.04], and relative *post-hoc* tests showed larger amplitude in the perceptual than imaginative condition, at central sites, for hot and pain but not cold images. In both conditions, LPP was larger to pain than hot stimuli and to the latter than cold stimuli.

##### 3.3.2.3. Affective states (feeling sadness, cheerfulness, fear)

The ANOVA performed on LPP amplitudes for this macro-category yielded the significance of Condition [*F*(1,19) = 4.95, *p* < 0.038], with larger amplitudes in the perceptual (2.14 μV, SE = 0.49) than imaginative condition (0.64 μV, SE = 0.49). Also significant was the interaction of Condition × Category × Electrode [*F*(2,38) = 6.05, *p* < 0.005]. Tukey *post-hoc* tests showed that, only at central sites (C1,C2), LPP was larger in the perceptual than imaginative conditions. Furthermore LPP was larger to fear (1.89 μV, SE = 0.66) than cheerful (0.24 μV, SD = 0.60) and sad stimuli (0.41 μV, SD = 0.69) during perception condition (see Figure 5B). Conversely LPP was greater to sad (3.02 μV, SE = 0.72) than fear images (1.98 μV, SE = 0.56) in the imaginative condition. The ANOVA also yielded the significance of Hemisphere factor [*F*(1,19) = 6.79, *p* < 0.05] indicating larger LPP amplitudes over the right (1.77 μV, SE = 0.42) than left hemisphere (1.01 μV, SE = 0.34) for both conditions.

##### 3.3.2.4. Secondary needs (music listening, moving, and playing)

The ANOVA performed on LPP amplitude for this macro-category yielded a significant interaction of Category × Electrode [*F*(2,38) = 3.89, *p* < 0.05]. *Post hoc* comparisons showed, at central sites (C1, C2) larger LPP amplitudes in response to music (2.36 μV, SE = 0.62) than playing (1.26 μV, SE = 0.50), regardless of condition. The significance of hemisphere factor [*F*(1,19) = 6.79, *p* < 0.05] indicated larger LPP over the right (2.01 μV, SE = 0.48) than left hemisphere (1.24 μV, SE = 0.37). The effect of Condition was not significant (*p* = 0.44).

### 3.4. Correlation between ERPs and imageability values

**FIGURE 6:**
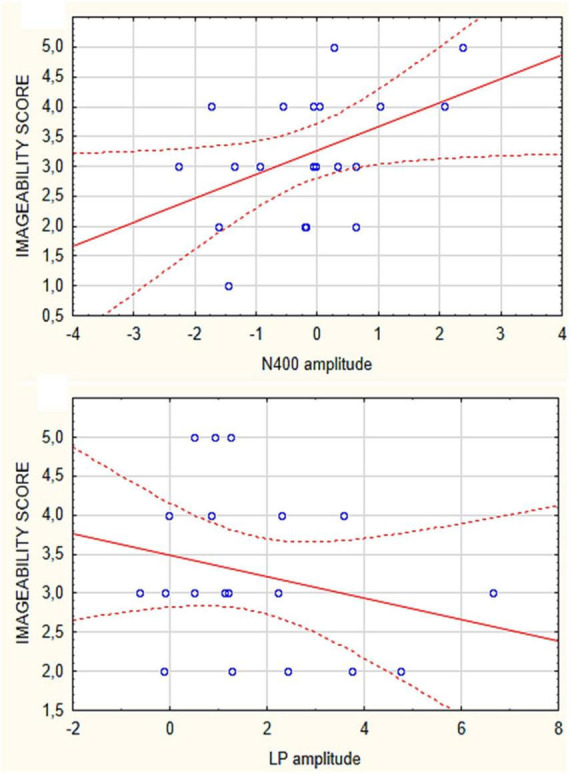
Spearman Rho correlations between imageability scores attributed to the secondary need category (music listening, moving, social play) and the mean areas values of N400 **(top)** and LPP responses **(bottom)** recorded in the individual participants.

Correlation analyses performed between the imageability scores attributed to the various micro-categories of motivational states and the amplitudes of N400 and LP were significant only for secondary needs. Both N400 (Spearman Rho = 0.45) and LP (Spearman Rho = 0.32) correlated in amplitude with imageability of stimuli relative to: “desire to listen to music,” “desire to play video games” and “desire to exercise” (as visible in Figure 6). The more difficult was the imagery task, the larger were ERP components. No significant correlation was found for the other needs. Spearman Rho correlations were also performed between imagery vs. perception related N400 amplitudes, which yielded the significance of rho = −3.3435 (*p* < 0.05). The correlation between imagery and perception for LPP response did not appear to be significant.

### 3.5. Source reconstruction

The correlation between imageability values and N400 (400–600 ms) and LPP (800–1000 ms) amplitudes showed that secondary desires, not easier to imagine, nevertheless reflected a closer correspondence with evoked potentials generated at neocortical level than visceral sensations (presumably involving thalamus, hypothalamus, etc.) and other needs did. They were then selected (particularly the categories “desire to move” and “listening to music”) for source reconstruction based on the hypothesis that imagining music and movement activated distinctive cortical regions that could be traced with swLORETA.

**FIGURE 7:**
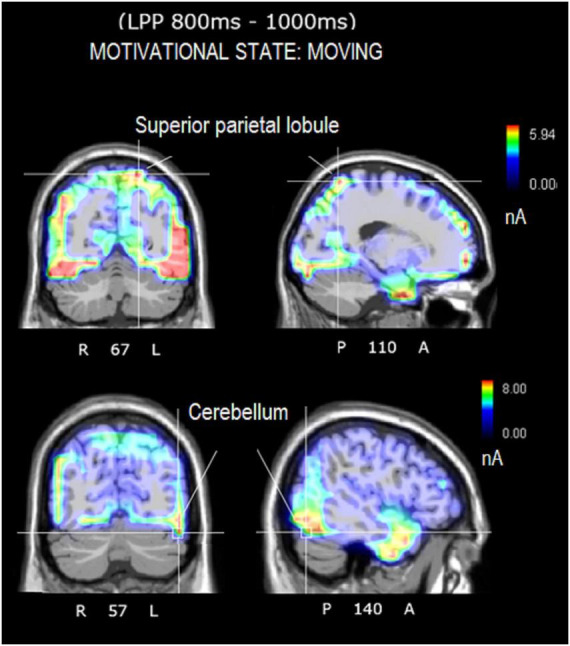
Axial, sagittal, and coronal brain sections showing the location and strength of electromagnetic dipoles explaining the surface LPP potential during the desire-to-move condition. Note that participants had to remain absolutely still and motionless during EEG recording.

**FIGURE 8:**
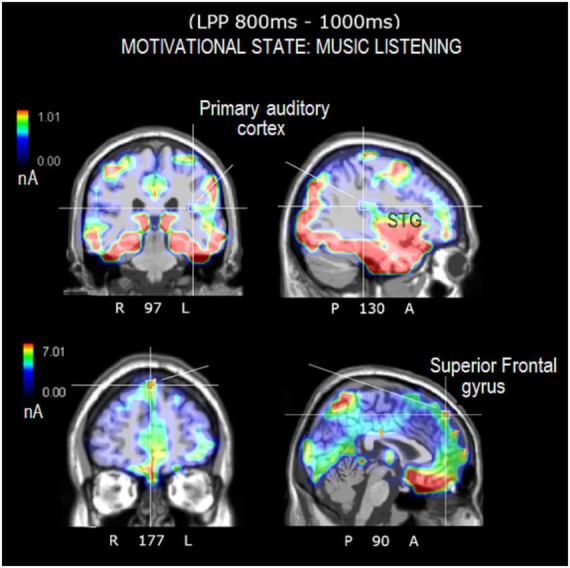
Axial, sagittal, and coronal brain sections showing the location and strength of electromagnetic dipoles explaining the surface LPP potential during the music-listening desire condition. Note that the participants were in complete silence inside an anechoic chamber during EEG recording.

The source reconstruction showed the strong activation of the bilateral superior frontal gyrus (SFG) in all the conditions, thus suggesting its being at the basis of imagery processes (magnitudes were, for MUSIC: (N400) left BA6 = 8.17, left BA = 10 6.89, right BA10 = 6.56. (LPP) left BA6 = 9.19, right BA = 10 9.12, left BA6 = 8.55. For MOVEMENT (N400) left BA10 = 7.07, right BA6 = 6.25, right BA6 = 0.11. (LPP) left BA10 = 8.53, right BA10 = 6.54, left BA6 = 4.55). In general, the superior frontal gyrus (SFG) would contribute to higher cognitive functions and particularly to working memory (du Boisgueheneuc et al., 2006). Table 3 lists the other dipoles found to be significantly active during the imagination of “desire to move” and “to listen to music.” It can be noted how brain activation was quite distinctive and specialized, across imagery categories. Figure 7 illustrates the intracranial generators of surface electric potentials relative to the desire to move at LP level, and including the left cerebellum involved in simulated movement (e.g., Naito et al., 2002) and the left superior parietal lobule involved in action representation. The strongest neural source identified in response to imagery in “music desire” category, with 9.43 nA of magnitude was represented by the right Superior Frontal Gyrus (BA8), typically active during music listening and creation. A second generator was the Superior Temporal Gyrus (BA41) of the left hemisphere, where Heschl’s gyri are located (primary auditory area A1). Both generators are clearly visible in the neuroimages of Figure 8.

**TABLE 3:** List of active electromagnetic dipoles explaining the surface voltage of N400 and LP surface potentials according to swLORETA.

Magn.	Hem.	Area	BA	Presumed function
Music listening				
**N400 (400–600 ms)**				
7.39	L	Middle frontal gyrus	46	Music creativity/imagery
7.1	R	Superior frontal gyrus	8	Music listening
7.96	L	Superior parietal lobule	7	Imagery
7.64	R	Supramarginal gyrus	40	
0.13	R	Superior temporal gyrus	38	Auditory processing
0.12	L	Inferior temporal gyrus	20	
0.12	L	Uncus	36	Music affective processing
**LP (600–800 ms)**				
9.43	R	Superior frontal gyrus	8	Music listening
8.98	R	Medial frontal gyrus	9	Music creativity/imagery
6.33	L	Superior temporal gyrus	41	Primary auditory area (A1, Heschl Gyri)
7.3	R	Cingulate gyrus	23	Music affective processing
0.16	R	Superior temporalo gyrus	38	Auditory processing
0.15	R	Middle temporal gyrus	21	Imagery
**Moving**				
**N400 (400–600 ms)**				
8.71	L	Superior parietal lobule	7	Kinesthetic imagery
6.74	L	Middle frontal gyrus	9	
5.97	L	Supramarginal gyrus	40	Action processing
0.18	R	Cerebellum/fusiform gyrus		Simulated movement/motor imagery
0.12	L	Cerebellum, posterior lobe,		Simulated movement/motor imagery
**LP (600–800 ms)**				
8.32	L	Cerebellum, posterior lobe		Simulated movement/motor imagery
7.98	R	Fusiform gyrus	20	Visual mental imagery
7.53	L	Middle temporal gyrus	21	
6.49	L	Superior parietal lobule	7	Kinesthetic imagery
5.07	L	Supramarginal gyrus	40	Somatosensory/action processing

Magn, magnitude; Hem, hemisphere; BA, Brodmann area. The rightmost column describes the presumed brain function according to available neuroimaging literature.

### 3.6. A comparison of the 12 microstates (in a BCI perspective)

**FIGURE 9:**
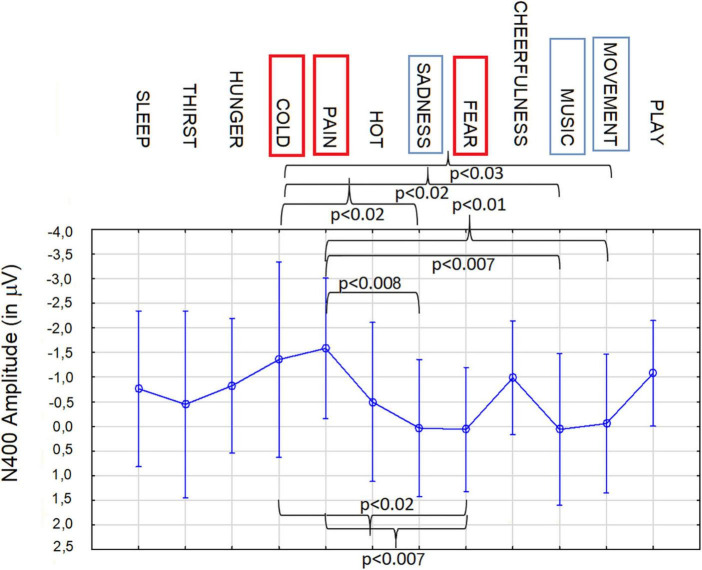
Mean area amplitude values of N400 responses recorded at AF3 and AF4 sites in the 400–600 ms time window, during imagination, as a function of the need category. Anterior N400 appears to be a reliable neuroelectric marker, in showing significant differences in magnitude depending on the valence and the sensory, emotional, or visceral nature of the motivational/physiological imaginal state.

Two further ANOVAs (N400 and LPP) were performed to compare all 12 motivational states in the imagery condition for BCI purposes. The ANOVA carried out on N400 amplitude values showed the significance of category factor [*F*(11, 209) = 1.8, *p* < 0.05]. *Post hoc* comparisons showed that, N400 to imagined pain (−1.58 μV, SE = 0.48) differed from N400 to imagined desire for movement (−0.05 μV, SE = 0.48; *p* < 0.01), music listening (−0.065 μV, SE = 0.52; *p* = 0.007), fear state (0.064 μV, SE = 0.43; *p* < 0.007), sadness state (0.038 μV, SE = 0.47; *p* = 0.008), and tended to be different from N400 to imagined thirst (−0.44 μV, SE = 0.64; *p* = 0.06) and hot states (−0.49 μV, SE = 0.54; *p* = 0.07), as can be observed in Figure 9. In addition N400 to imagined cold (−1.36 μV, SE = 0.67) differed from N400 to imagined desire for movement (−0.05 μV, SE = 0.48; *p* < 0.03), music listening (−0.065 μV, SE = 0.52; *p* < 0.02), fear (0.064 μV, SE = 0.43; *p* < 0.02) and sadness states (0.038 μV, SE = 0.47; *p* < 0.02). N400 to imagined fear (0.064 μV, SE = 0.43) differed from N400 to imagined pain (−1.58 μV, SE = 0.48; *p* = 0.007) and cold states (1.36 μV, SE = 0.67; *p* < 0.02) and tended to differ from N400 to imagined desire to play (−1.08 *p* = 0.06). As detailed above, N400 to imagined movement, music listening, fear and sadness states significantly differed from N400 to pain and cold imagined states. N400 to imagined desire to play (−1.08 μV, SE = 0.36) tended to differ from N400 to imagined music listening (*p* = 0.06), fear (*p* = 0.06) and sadness states (*p* = 0.06).

It can be observed how anterior frontal N400 recorded in the 400–600 ms time window acted as a reliable marker of life threatening states, such as cold, pain and fear, but also, to a lesser degree, sadness, and secondary needs (i.e., the desire to move, listen to music, and play with friends).

Conversely, the ANOVA carried out on LPP amplitudes in the imagery condition did not reveal a significance for the category factor [*F*(11, 209) = 0.804, *p* = 0.635], in this specific statistical design.

## 4. Discussion

The aim of the present study was to investigate the neural correlates associated with the perception and imagination of motivational states, desires and craves. EEG/ERP signals were recorded during an experimental task comprising the visual presentation of pictograms depicting a certain need, followed by probes (empty yellow frames) that prompted the imagination of the specific motivational state. If reliable markers of motivational or imaginative processes could be recorded, this would allow their use in the field of BCI. While some EEG studies involving imagining auditory (Lee et al., 2019) and visual (Bobrov et al., 2011) stimuli demonstrate this possibility, in contrast, still little evidence in the literature shows the capability of classifying EEG signals reflecting different categories of induced mental and emotional states. For example, one very interesting study (Fan et al., 2018) was able to classify mental states such as engagement, boredom, amusement, frustration, and workload by analyzing EEG frequency and power.

In this study, twelve micro-categories of imagined needs, divided into four macro-categories (primary visceral needs, somatosensory sensations, affective states and secondary needs) were considered as most relevant for a possible BCI usage and compared in the perceptual and imaginative condition. The macro-category analyses showed that ERPs (especially anterior/frontal N400) were larger in the perceptual than imaginative condition, except for somatosensory sensations. This finding fully agrees with previous neuroimaging evidence describing imagination as a kind of weaker and noisier perceptual experience (Pearson, 2019), as opposed to the more vivid and detailed sensorial experience (Dijkstra et al., 2017). Indeed, a fMRI study found a correlation between the vividness of imagery and the similarity of BOLD responses in early visual cortex across perception and imagery conditions (Lee et al., 2012). Consistent evidences of smaller potentials during imagination than perception were reported in previous ERP studies on perceived and imagined emotional and food real-life photos (e.g., Marmolejo-Ramos et al., 2015) and on perceived or imagined auditory and visual objects (such as words, speech or music, Proverbio et al., 2023).

In this study, in the perception condition N400 was of similar amplitude across categories (possibly reflecting the perceptual matching of pictograms), but it was significantly larger during imagination of pain, hot and cold sensations than other imagined states. LPP amplitude did not differ as a function of Condition (except for somatosensory sensations), thus suggesting a similar pattern of brain activation across imagery and perception for the late-latency conceptual representation of needs. However, LPP was of greater amplitude over central sites during imagination and temporo/parietal ones during perception. Somewhat similarly, it was found that LPP was larger anteriorly during imagination and more posteriorly during perception, in studies investigating imagery for affective faces (Suess and Abdel Rahman, 2015), and for auditory/visual objects (Proverbio et al., 2023). Interestingly, hippocampal activity, crucial in the recall of representations from long-term memory (Kosslyn and Thompson, 2003) and a relevant component of imaginative activity, has been shown to be best picked up at central medial sites and central electrodes are long known to be optimal sites for motor imagery (Choi et al., 2006).

The present data also showed a right hemispheric asymmetry in the distribution of LPP, regardless of Condition (except that for somatosensory sensations, that elicited bilateral markers). The lateralization to the right scalp sites during both perception and imagination reflects a similarity between the activation of late-latency mental representation across the two conditions. However the scalp distribution does not necessarily reflect the activity of right inner brain structures, and no clear evidence exist of a right hemispheric dominance for mental imagery (Ehrlichman and Barrett, 1983). For instance, according to Liu et al. (2022) the left temporal lobe would be very active in visual mental imagery, but the asymmetry would depend on the mental contact, for example it would be left sided for orthographic material or right-sided for faces.

Anterior N400 data in the imagery condition revealed - as in the perception condition – a greater negativity over anterior frontal sites in response to stimuli of all macro-categories. However, it was larger during imagination of somatosensory sensations. This increased anterior amplitude may reflect a parallelism with the perception of sensory stimuli of heat, cold and pain, for which anterior functional activations have been previously demonstrated (Casey et al., 1996; Davis et al., 1998). It is very interesting to observe that somatosensory sensations eliciting N400 larger potentials in the imaginative conditions were also associated to high imageability scores (although smaller than visceral sensations), possibly indicating a certain ease or familiarity of subjects in feeling thermal and pain sensations.

N400 data revealed a greater average amplitude for the “playing” than “music listening” and “movement” secondary needs, regardless of condition. Given the sociality and enjoyment components associated with this typical juvenile drive, the larger activation might possibly reflect a stronger activity of the nigrostriatal orbitofrontal circuit, linked to the reinforcement and reward components characteristic of social playing. As for the affective states, regardless of condition, the N400 was of greater amplitude in response to positive states (such as cheerfulness) than negative ones (such as fear or sadness). The smaller electrical responses recorded during simulation of negative mental states, also found by Marmolejo-Ramos et al. (2015), was interpreted as reflecting a suppression/inhibition of unpleasant mental contents (Huang et al., 2021). Somewhat similarly, Ogawa and Nittono (2019) found smaller N400 amplitudes in response to imagined words under negative than positive mood. Regarding visceral primary needs, N400 was greater in response to hunger than to the desire to sleep; other studies have found hunger to be one of the most intense desire states, associated with clear and vivid imaginative states (Bradley and Moorey, 1988) and major autonomic activations. In fact, visceral sensations (hunger, thirst and sleep) were associated with the highest imageability scores. It is possible that they are the most frequently felt and common craving experiences, therefore more easily and vividly recalled by participants. According to Bradley and Moorey (1988) the stronger craves (e.g., drug craving for addicted individuals) are associated with particularly vivid imagining. Nevertheless our data showed that N400 and LPP electrical signals most closely correlated (in amplitude) with imageability scores were reflected by secondary needs (play, music and movement), apparently, not very easy to imagine. A possible interpretation for this inconsistency might concern the almost entirely neocortical (vs. visceral) nature of these mental representations, closer to volition than to instinctual and irrepressible needs. It should be considered that ERPs are the sum of post-synaptic excitatory and inhibitory potentials of neurons located within the cortex layers (Zani and Proverbio, 2003), somewhat picking also some activity from the hippocampus and the amygdala, but hardly reflecting hypothalamic signals. On the other hand, visceral needs would be more “reptilian”: the “satiety center” being located in the ventromedial hypothalamus and the “feeding center” in the lateral hypothalamus (Ahima and Antwi, 2008). A brain structure called lamina terminalis (LT) in the mammalian forebrain would primarily regulates thirst, and particularly the median preoptic nucleus (Augustine et al., 2020). Another main difference between primary/homeostatic sensations and secondary needs is their automaticity and their (partial) lack of cognitive control, whereas the latter would be associated to decision making and volition, and thus with prefrontal processing (Liljenström, 2022).

Perhaps the most insightful piece of data was the comparison of ERP responses elicited during imagery of all 12 microstates, which represents the closest approximation to a possible use of this paradigm with BCI systems. LPP potential recorded at central sites (according to previous literature on imagery, e.g., Proverbio et al., 2023) did not seem to index reliably the different motivational states, possibly because of its late latency (800–1000 ms) and its cognitive, more a-specific nature. On the other hand, earlier N400 potential, recorded at anterior frontal sites between 400 and 600 ms, was significantly modulated by imaginative state. Its amplitude changed considerably during the most dramatic homeostatic and motivational states (namely, cold, pain and fear). Differences in N400 amplitude were also found for affective and secondary states. Peculiarly, anterior N400 was not modulated by visceral needs (hunger, thirst and sleep), as also shown by the micro-category analysis. Source reconstruction applied to secondary needs (i.e., music and movement) showed that N400 generators lied within cortical sensorimotor and prefrontal areas. Therefore, it is not surprising that visceral sensations (arising from hypothalamic structures such as the hunger and satiety centers, or the ventrolateral preoptic nucleus) would be “transmitted” less clearly by neocortical electrical potentials.

In detail, the source reconstruction applied to bioelectric potentials during movement-related imagery revealed significant left-sided activation of cerebellum/fusiform gyrus, posterior parietal lobule (BA7), and other sensorimotor areas. Parietal regions are implicated in movement representation as shown by literature on motor mental imagination as “emulation” or internal representation of movement (Wohlschläger, 2001; Oostra et al., 2016; Sakreida et al., 2018; Orlandi et al., 2020). It is interesting to note that brain damage to the fibers connecting the posterior parietal cortex to the dorsal premotor cortex would appear to impair motor mental imagery abilities (Oostra et al., 2016). In addition, the cerebellar involvement during the motor imagination of automatic and repeated movements such as walking, is well documented in the imagery literature (Decety et al., 1990; Guillot et al., 2009). Again, significant bilateral cerebellar activations were detected during a motor imagining task of an automatic and repeated movement of writing (Decety et al., 1990; Guillot et al., 2009). The results of source reconstruction are fully compatible with knowledge on motor imagery. Source reconstruction related to musical imagery, on the other hand, showed significant activation of the left primary auditory area (BA41, Heschl’s gyrus) and the right superior frontal gyrus (BA8), which are particularly involved in listening to and perceiving music (Halpern and Zatorre, 1999; Proverbio and Piotti, 2021). For example, Halpern et al. (2004) found that the secondary auditory cortex was significantly active during timbre imagery and perception. Other PET studies investigating imagery of music melody found an involvement of the right superior temporal gyrus, of the posterior parietal cortex as well as the right prefrontal lobe (Halpern and Zatorre, 1999).

For both types of mental simulations, the prefrontal and superior frontal cortices, which are known to support short-term memory mechanisms underlying the ability to activate mental images and the maintenance and mental manipulation of information, were found to be particularly active (Chen et al., 2021). In addition, the activation of visual areas, such as the fusiform gyrus, during motor imagery fits with previous literature on *visual motor imagery* (Annett, 1995) dealing with visuospatial (as opposed to kinesthetic), aspects of imagined movement (Decety, 1996; Jiang et al., 2015). At this regard, Spagna et al. (2021) showed the key role of the fusiform gyrus in visual mental imagery.

The activations identified by these initial analyses appear to be in line with the literature on imagining motivational states and craves. The further development would be the use of BCI systems. Quite recently, Koban et al. (2022) used machine learning to identify a set of cross-validated fMRI neuromarkers that predicted self-reported intensity of cue-induced drug and food craving (defined *Neurobiological Craving Signature).* They found a circuit of key regions including the ventromedial prefrontal and cingulate cortices, ventral striatum, temporal/parietal association areas, mediodorsal thalamus and cerebellum, many of which were found active in our study, except those deeper and farther from the neocortex. Overall, the present results seem to have provided interesting markers (in particular, the anterior N400 and the central LPP), which appeared to be distinctive and informative. Their behavior changed specifically as a function of the various motivational macro- and micro-states. The data will be used by computational simulations to test the reliability of the markers for BCI purposes.

### 4.1. Study limits and future perspectives

One of the potential limits of the present experimental paradigm is that, notwithstanding the presence of a post-experiment questionnaire ensuring that subjects paid close attention throughout the experiment, no on-line control of the task execution was possible. Future experiments might tests alternative paradigms involving trial-by-trial responses to probes.

In addition, it would be interesting to perform in the future an analysis of the perceived arousal degree related to the different microstates of the Pictionary to help discussing the differences in the gradient of evoked potentials. It would also be desirable to perform a source reconstruction for all microstates and the two ERP components, but this would require a later study because of the extensive amount of data to be examined and discussed. The possibility of using current data for effective classification through an A.I. deep learning system is also being investigated in our laboratory, and should be further explored.

## Data availability statement

The original contributions presented in this study are included in the article/supplementary material, further inquiries can be directed to the corresponding author.

## Ethics statement

The studies involving human participants were reviewed and approved by the Ethical Committee of University of Milano-Bicocca. The patients/participants provided their written informed consent to participate in this study.

## Author contributions

AP conceived and planned the experiments and took the lead in writing the manuscript. FP carried out the experiments and prepared and validated the stimulus material. AP and FP analyzed the data and contributed to the interpretation of the results. Both authors provided the critical feedback and helped to shape the research, analysis, and manuscript.
